# Triple Immunotherapy Overcomes Immune Evasion by Tumor in a Melanoma Mouse Model

**DOI:** 10.3389/fonc.2020.00839

**Published:** 2020-06-12

**Authors:** Mary-Ann N. Jallad, Abdo R. Jurjus, Elias A. Rahal, Alexander M. Abdelnoor

**Affiliations:** ^1^Department of Experimental Pathology, Immunology and Microbiology, Faculty of Medicine, American University of Beirut, Beirut, Lebanon; ^2^Department of Anatomy, Cell Biology and Physiological Sciences, Faculty of Medicine, American University of Beirut, Beirut, Lebanon

**Keywords:** melanoma, immunotherapy, anti-CTLA-4 antibodies, MPLA, IDO, 1-MT

## Abstract

**Background:** Melanoma is a malignancy with increasing incidence that underlies most skin cancer-related deaths. Advanced melanoma patients still have poor prognosis despite recently developed immunotherapies. This study devises a triple immunotherapy to treat melanoma in a mouse model. The combination includes anti-cytotoxic T-lymphocyte-associated protein 4 (CTLA4) antibodies, Monophosphoryl-lipid-A (MPLA), and an Indolamine-Dioxygenase-1 (IDO1) inhibitor. The aim of the study is, first, to rule out any major toxic effects related to this therapy and, second, to assess its antitumor effects.

**Methods:** Cancer-free C57BL/6 mice were randomized into control groups and groups receiving single, dual, or triple therapies of the defined treatments. Clinical signs, weight gain, and histological sections from their main organs were assessed. Then, melanoma-bearing mice were segregated into similar groups, monitored for survival, and their tumor size was measured repeatedly. Finally, flow cytometry was used to analyze immune cell populations in the tumor masses including CD4+, CD8+, and regulatory T cells in addition to natural killer cells.

**Results:** No adverse effects were detected in any of the treated groups. Survival analysis indicated that the groups receiving dual or triple therapies had prolonged survival compared to the controls. However, the group receiving triple therapy was the only group to show statistically significant increase in survival compared to the controls. Tumor size progression paralleled the survival outcome. The group receiving the triple therapy showed statistically significant smaller tumor sizes compared to all the other groups throughout the whole monitoring period. Flow cytometry used to analyze immune cell populations in the tumor mass indicated that the triple immune therapy was capable of significantly enhancing the natural killer cell counts as well as the CD3+CD4+/Treg and CD3+CD8+/Treg ratios possibly enhancing the anti-tumorigenic environment.

**Conclusions:** Generated data rule out any major adverse events pertaining to the triple immunotherapy and reveal its enhanced effectiveness in thwarting melanoma progression over all other tested treatments.

## Introduction

Melanoma is classified among the most aggressive tumors and accounts for the majority of deaths related to skin cancer. Considerable research efforts have hence been put into investigating treatment approaches yielding substantial advancements in this field as of 2011. This progress was mostly achieved through the implementation of immunotherapies (1–3). Those that revolutionized the treatment of advanced melanoma and which have been approved by the European Medicines Agency (EMA) and the Food and Drug Administration (FDA) are mainly checkpoint inhibitors including anti-cytotoxic T-lymphocyte-associated protein 4 (CTLA-4) and anti-Programmed cell death protein 1 (PD1), and oncolytic viruses such as T-vec (1, 2).

In spite of this progress, the low survival rates of melanoma patients to date are still quite alarming. Recent studies showed that a promising endeavor to overcome this challenge is to use combination immunotherapies. Yet, substantial work lies ahead to determine therapeutic combinations that are more effective and which would improve the prognosis of advanced melanoma patients (4–7).

In this study, a triple combination of immunotherapies was devised, and it included the anti-CTLA-4 monoclonal antibody, which was the first FDA-approved immune-checkpoint blocker for the treatment of melanoma. This inhibitory agent stops CTLA-4 from inactivating T cells, therefore enhancing the activity of effector T cells against tumor cells (8). The resulting maintenance of antitumor adaptive immunity can be clinically significant (9), yet this approach does not include any enhancement of the innate immune responses that could largely improve the therapeutic outcome. Microbial products are effective modulators of host responses (10, 11), consequently, the second component of the triple immunotherapy is Monophosphoryl-Lipid-A (MPLA), which is a potent activator of innate immune responses. In 1978, it was reported that post-lipopolysaccharide mouse sera conferred resistance to the TA3-Ha mouse tumor. Moreover, it was shown that the polysaccharide segment (PS now named MPLA) of lipopolysaccharide possessed antitumor activity (12, 13). Regardless of its antitumor effect, MPLA is now used as a non-toxic adjuvant with anti-cancer agents (14, 15). MPLA binds to Toll-like receptor 4 (TLR4), leading to the production of type I interferons and the secretion of antitumor cytokines (14–16). This treatment not only activates the innate immune responses but also promotes adaptive immunity while inhibiting regulatory T cells (4, 17, 18). However, treatments with anti-CTLA-4, MPLA, or both lead to the upregulated production of the immune-suppressive enzyme Indolamine-Dioxygenase-1 (IDO-1) (19–23). Therefore, the third component of the proposed combination is 1-methyl-tryptophan (1-MT) which is an IDO-1 inhibitor. Normally, an increase in IDO-1 results, first, in the depletion of tryptophan, therefore contributing to the expansion of Tregs, and second, to an increase in the tryptophan pathway metabolites, therefore suppressing adaptive T cell immunity (21, 24–26). Hence, the inhibition of IDO-1 will deter the immune suppression, thus enhancing the likelihood of an adequate antitumor immune response (21). Accordingly, this combination is expected to hinder the ability of cancer cells to evade the immune system.

The main purpose of this study was hence to rule out any major adverse events pertaining to the proposed triple immunotherapy and to assess its antitumor effects in comparison with single and dual combinations of its components. The results positively demonstrated higher effectiveness of the triple combination in increasing animal survival and thwarting tumor progression in melanoma-bearing mice.

## Materials and Methods

### Mice

All mice used were female C57BL/6 mice aged 8–10 weeks old, weighing 20–22 g each. The experiments were conducted according to the regulations of the Institutional Animal Care and Use Committee at the American University of Beirut.

### Treatment Agents

MPLA was obtained from In VivoGen, Toulouse, France; CTLA-4ab was obtained from Bioxcell, West Lebanon, NH; and 1-MT was obtained from Sigma Aldrich/Merck, Darmstadt, Germany.

### Monitoring Adverse Effects

To ascertain that none of the three agents alone or in combination were toxic to C57BL/6 mice, nine groups of three mice each were used and treated as follows: group 1 was an untreated control; group 2 was a saline-treated control (saline being the vehicle of all used treatments); groups 3, 4, and 5 were treated with single therapies of either MPLA, anti-CTLA4-antibodies, or 1-MT; groups 6, 7, and 8 were treated with dual therapies of these treatments; and group 9 was treated with all three immunotherapeutic agents. Doses were as follows: 10 μg MPLA was administered subcutaneously into the upper right flank on day 8 and then on day 15. As for anti-CTLA4, 200 μg was given intraperitoneally at day 3 and 100 μg was given on days 6, 9, 12, and 15. 1-MT was given in daily intraperitoneal doses of 2.25 mg. All mice were monitored throughout the treatment period and for the following 3 months. The monitoring included observation of clinical signs such as the grooming of the fur, mobility, hunched posture, respiratory distress, presence/consistency of stools and failure to eat, as well as weekly weight measurement. At the end of the monitoring period, the mice were sacrificed and histological evaluation of the liver, heart, kidneys, and lungs was performed.

### B16F10 Melanoma Mouse Model

Cells used for the tumor challenge were B16F10 melanoma cells, which are congeneic to the C57BL/6 mice. These cells were cultured in RPMI medium (Lonza, Basel, Switzerland) supplemented with 10% fetal bovine serum (Sigma-Aldrich/Merk, Darmstadt, Germany), 1% glutamine (Lonza, Basel, Switzerland), and 1% Pen-Strep (Lonza, Basel, Switzerland). The tumor model was generated by injecting 10^6^ melanoma cells subcutaneously into the upper right flank of mice at Day 0 of each experiment.

### Evaluating the Antitumor Effect

Mice were segregated into nine groups (containing 12 to 13 mice each) and treated as described above for monitoring adverse events but with MPLA being injected intratumorally. Survival was recorded and mice were monitored. Monitoring included daily observation of clinical signs as well as tumor measurements using a caliper every 3–4 days starting on day 10. Tumor volumes were determined using the following formula: Volume = π/6 (*LWW*), where *L* is the longest side measured and *W* is the shortest side measured. This procedure was conducted in two independent experiments.

### Assessment of Tumor-Infiltrating Immune Cells

Examining the tumor-infiltrating immune cells was performed as described previously by Pachynski et al. (27). Briefly, three mice from each of the nine groups were sacrificed on day 16 post-tumor inductions. Their tumor masses were excised and mechanically homogenized into cell suspensions using cell strainers. Cells were counted, fixed, and stained for detection of the CD4+ T cell population (using anti-CD3 and anti-CD4), the CD8+ T cell population (using anti-CD3 and anti-CD8), the Tregs (using anti-CD3, anti-CD4, and anti-CD25), and the NK cells (using anti-NK1.1), and analyzed by flow cytometry (BD FACSAria). Antibodies used were purchased from (Biolegend, San Diego, CA).

### Statistical Tests

Data were analyzed using GraphPad Prism. Two-way ANOVA was used to compare more than two groups. Tukey's and Dunnett's *post hoc* tests were used for multiple comparisons within groups. Kaplan–Meier was used for survival analysis, the outcomes were assessed by the Mantel–Cox log-rank test, and Bonferroni correction was used to determine significance. *P*-values less than 0.05 were considered statistically significant unless stated otherwise.

## Results

### Adverse Effects of MPLA, CTLA-4ab, and MT

**Figure 1:**
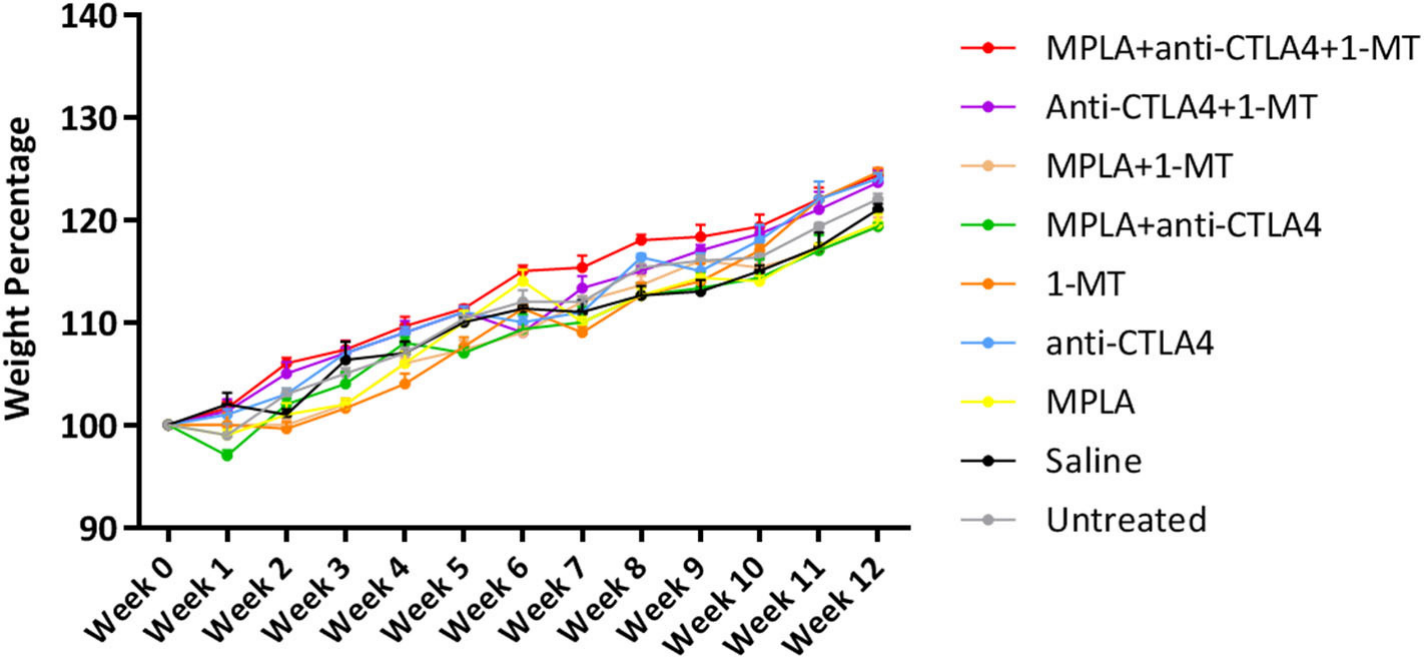
Average mouse weight per group of tumor-free C57BL/6 mice treated with MPLA, CTLA-4ab, 1-MT or their combinations (*n* = 3).

**Figure 2:**
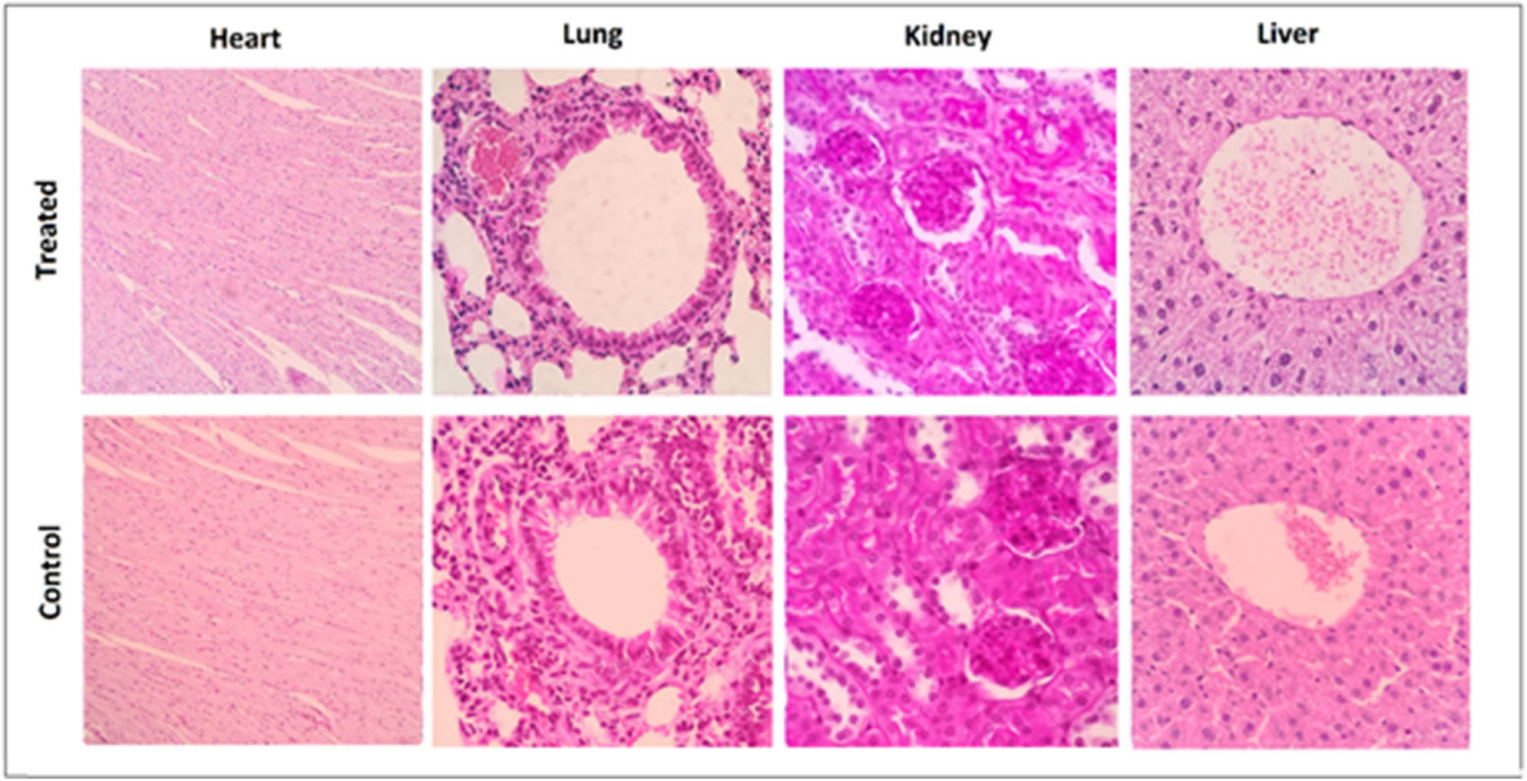
Representative histological sections taken at 3 months after completion of the treatment from tumor-free C57BL6 mice in the control group and the group receiving triple therapy (MPLA, CTLA-4ab, and 1-MT). Heart sections are presented at a 100× magnification while lung, kidney, and liver sections are presented at a 400× magnification (*n* = 3).

None of the three agents given alone or in combination caused adverse effects in mice. There was an increase in the weight of all mouse groups during the 12-week observation period (Figure 1). Moreover, no adverse clinical signs (including ataxia, lethargy, aggressiveness, etc.) were observed. Mice were sacrificed after 12 weeks, and their lungs, kidneys, heart, and liver had normal anatomical and histological features (Figure 2).

### The Effect of MPLA, CTLA-4ab, MT, and Their Combinations in the B16F10 Melanoma Mouse Model

**Figure 3:**
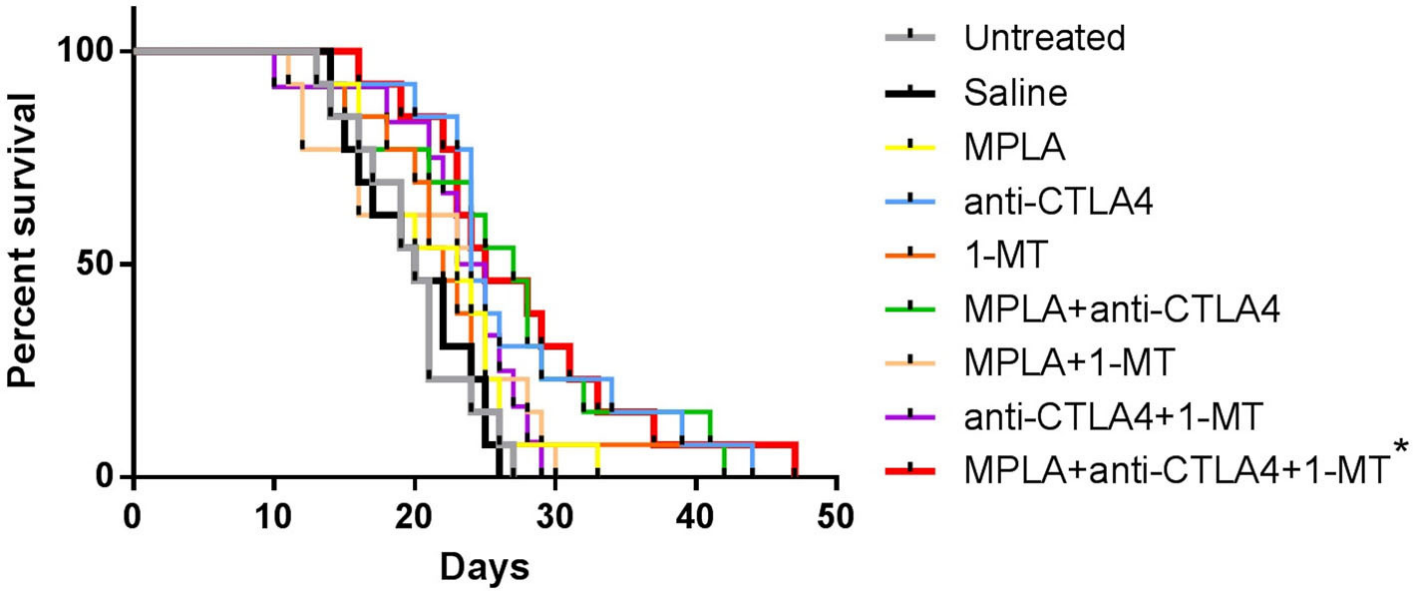
Percent survival of C57BL/6 mice following tumor induction with B16F10 melanoma cells and treatment with MPLA, CTLA-4ab, 1-MT or their combinations. Data represent two independent experiments (*n* = 12–13). *p < 0.005 compared to the untreated or saline-treated group.

Although some enhanced survival was observed with the single agent treatments, such as 1-MT and CTLA-4ab, and with some combinations, such as MPLA + CTLA-4ab, the enhanced survival was not statistically significant compared to the untreated or saline-treated groups. The only combination to cause a statistically significant difference in survival when applying the Bonferroni correction was when mice were treated with a combination of the three agents (Figure 3).

**Figure 4:**
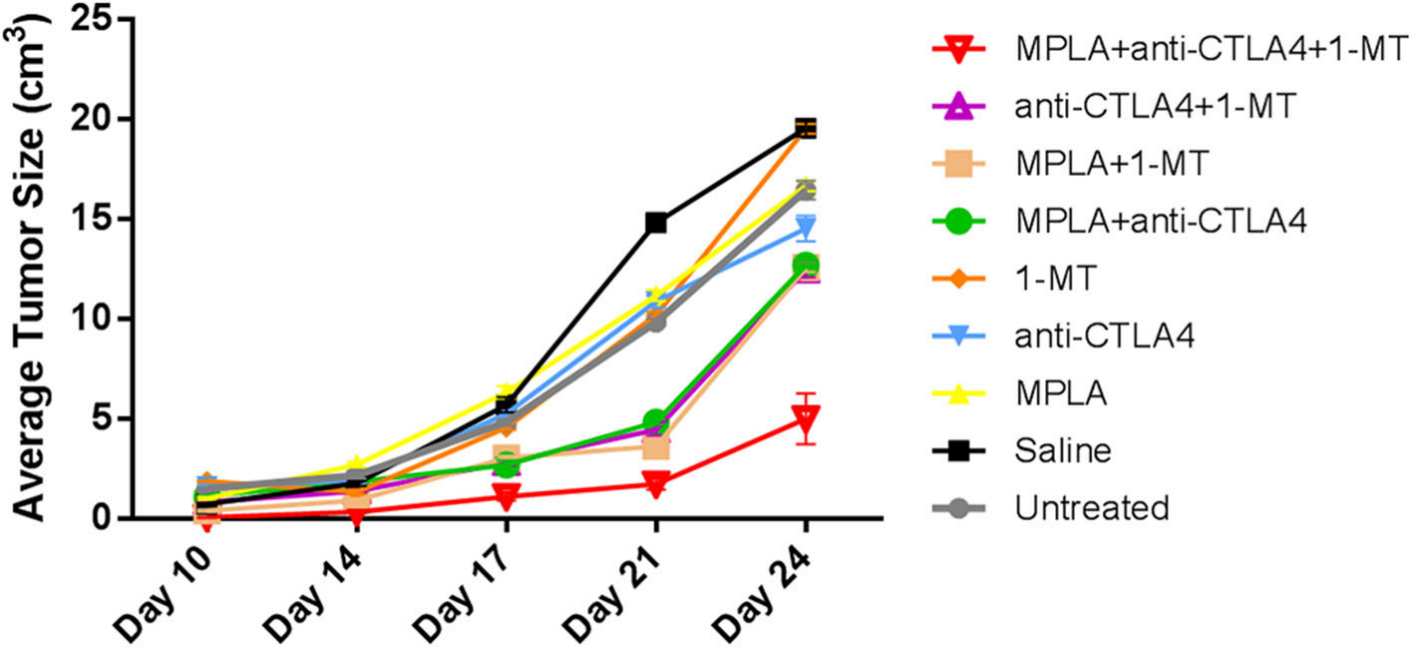
Average tumor sizes in C57BL/6 mice following tumor induction with B16F10 melanoma cells and treatment with MPLA, CTLA-4ab, 1-MT, or their combinations (*n* = 10). Numerical representations and statistical significance of tumor size variations are indicated in Table 1.

Tumor size progression assessment showed that although some combinations resulted in smaller tumor sizes compared to controls, the group of mice treated with the triple combination was the one to cause the greatest statistically significant reduction in tumor size; this reduction was by about 70% (Figure 4 and Table 1) by day 24 post-tumor induction.

**Table 1:** Average tumor sizes in C57BL/6 mice following tumor induction with B16F10 melanoma cells and treatment with MPLA, CTLA-4ab, 1-MT, or their combinations (*n* = 10).

**Treatment**	**Size of tumor (cm**^****3****^**) on day**				
	**10**	**14**	**17**	**21**	**24**
Untreated	1.49^*^	2.15^*^	4.81^*^	9.82^*^	16.43^*^
Saline	0.73^*^	1.75^*^	5.68^*^	14.79^*^	19.53^*^
MPLA	1.00^*^	2.70^*^	6.31^*^	11.15^*^	16.63^*^
CTLA-4ab	1.59^*^	1.92^*^	5.23^*^	10.86^*^	14.51^*^
MT	1.85^*^	1.33^*^	4.56^*^	10.17^*^	19.51^*^
MPLA + CTLA-4ab	1.11^*^	1.85^*^	2.68^*^	4.83^*^	12.68^*^
MPLA + MT	0.39^*^	0.91^*^	3.02^*^	3.62^*^	12.56^*^
CTLA-4ab + MT	0.82^*^	1.36^*^	2.81^*^	4.45^*^	12.44^*^
MPLA + CTLA-4ab + MT	0.06	0.33	1.10	1.72	4.98

* *p < 0.05 compared to the MPLA + CTLA-4ab + 1-MT-treated group*.

### Assessment of the Tumor-Infiltrating Immune Cells

**Figure 5:**
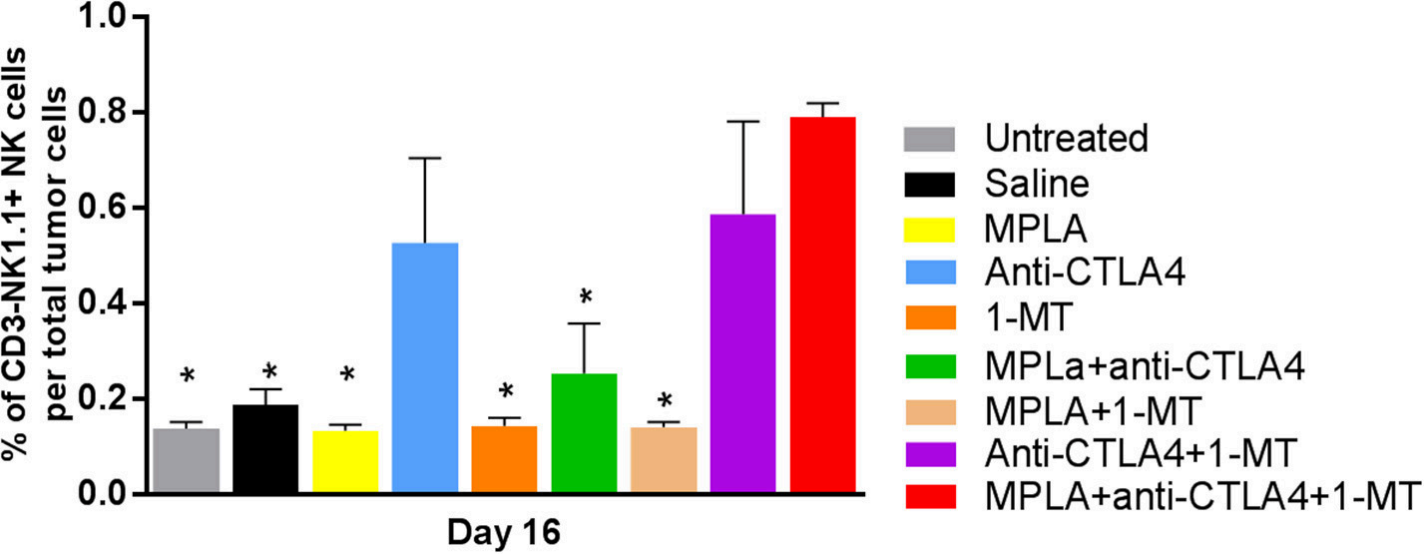
Percent of NK cells per tumor in the B16F10 melanoma mouse model treated with various immunotherapeutic regimens (*n* = 3). *p < 0.05 compared to the MPLA + CTLA-4ab + 1-MT-treated group.

**Figure 6:**
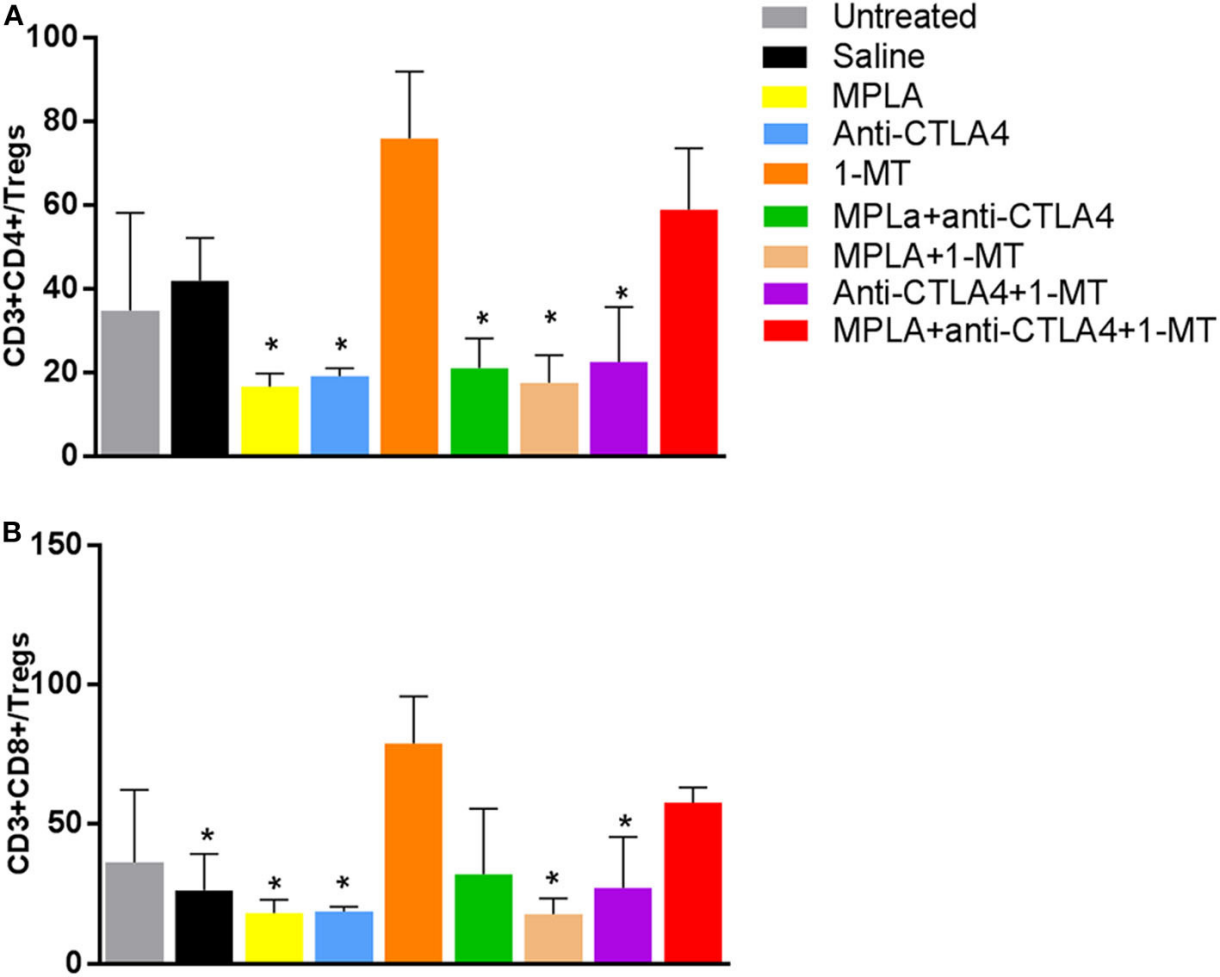
**(A)** Ratio of CD4+ T cells to Tregs per tumor and **(B)** ratio of CD8+ T cells to Tregs per tumor in the B16F10 melanoma mouse model treated with various immunotherapeutic regimens (*n* = 3). *p < 0.05 compared to the MPLA + CTLA-4ab + 1-MT-treated group.

Regulatory T cells had the lowest level in the group treated with the triple combination among all the tested groups except the 1-MT group. This group also had the highest levels of NK cells among all groups (Figure 5). Moreover, while the absolute numbers of CD3+CD4+ and CD3+CD8+ cells showed little significant changes among the various groups, the ratios of the numbers of these cells to Tregs did. The ratios of CD3+CD8+ cells to Tregs and CD3+CD4+ cells to Tregs were highest in the group receiving the triple immunotherapy compared to all groups with the exception of the group receiving 1-MT (Figure 6).

## Discussion

The rationale behind the selection of the three immunotherapeutic agents for the combination assessed in this study was that such a combination provides sufficient elements to instigate both the innate and the adaptive immune systems; moreover, their effects were expected to be maintained long enough as a result of inhibition of immunosuppressive mechanisms. However, the main concern of this approach was the risk of toxicity thus resulting in deleterious consequences. The absence of abnormal clinical signs and the comparable weight gain rates between all treatment groups and the control group imply that no major adverse events result from any of the single, dual, or triple therapies tested. This interpretation is further verified by examination of the histological sections from the heart, lungs, liver, and kidneys, which showed normal anatomical and histological features for all experimental groups.

It is worth noting that the B16F10 cells used to generate the melanoma mouse model in this study induce a very severe type of tumor that is notoriously difficult to treat compared to other cell lines used in the development of murine melanoma models (28). In addition, the number of cells we injected to induce tumors in mice is the highest reported in the literature for this model and is 15 to 20 times the minimum tumorigenic dose (29). The purpose of using such a high number of cells to initiate cancer is to validate the effect of the tested therapeutic agents in treatment rather than prevention of tumors. The resulting model is therefore expected to provide better representation of what happens in the clinic whereby patients only seek treatment after establishment of the disease or even at late stages of its progression. This approach aims at minimizing the gap often seen between the promising results of preclinical studies and the much less favorable outcomes in the subsequent clinical studies.

The tumor progression and survival outcomes validated the initial assumption of this study by showing an advantage to the triple immunotherapy over all other tested treatments. The group treated with anti-CTLA-4 showed the most improved outcomes among the single therapy groups which was expected since both MPLA and IDO inhibitors are used as treatment adjuvants as opposed to anti-CTLA-4, which is approved for treatment of a subset of melanoma patients as a single therapy (2, 30). Dual therapies showed slightly prolonged survival rates and delayed tumor progression but were outweighed by the results of the triple therapy. The speculated mechanisms underlying this outcome are an adequate activation of innate immunity by MPLA and a sufficient inhibition of tryptophan metabolism by 1-MT, which both complemented the anti-CTLA-4 effect of maintaining effector T cell activity.

Several combinations with anti-CTLA-4 have been reported in the literature. One of these combinations is that of anti-CTLA-4 with IDO-1 inhibitors. Studies have shown that this combination leads to an enhanced survival when compared to treatment with anti-CTLA-4 alone (5). This effect was not seen in the present study. Such a discrepancy may be due to the difference in the severity of the tumor model, in treatment doses and the modes of administration. Nevertheless, this emphasizes the effectiveness of the triple therapy, which, despite the severity of the model, had a significant effect in extending survival and thwarting tumor growth in comparison to all treated groups throughout the monitoring period.

Another studied combination is that of anti-CTLA-4 with anti-PD1, which was approved for the treatment of melanomas that do not express PD-1 (2, 6, 31, 32). It has been demonstrated that this kind of combination has an additive therapeutic effect compared to treatments with either anti-CTLA-4 or anti-PD1 as it impedes tumors more efficiently from evading immune responses; however, the response is dependent on the level of expression of PD-1 by tumor cells and therefore cannot be considered as standard treatment for all melanoma patients (33–35). Other tested combinations include the use of anti-CTLA-4 along with chemotherapeutic agents such as Imatinib or Dacarbazine, which have shown a superior effect to chemotherapies used alone but still with a limited success (36, 37). As opposed to these combinations, the advantage of the currently proposed triple therapy is that it uses agents with modes of actions that are not dependent on the genetic characterization of the melanoma or on the level of expression of certain markers, such as PD-1, by the tumor cells and therefore could be employed to treat a larger proportion of melanoma patients with an otherwise poor prognosis.

Examination of the tumor-infiltrating cell populations showed a significant increase of NK cells in the triple immunotherapy group and a significant decrease of Tregs. The triple immune therapy resulted in a decreased number of Tregs that was sufficient to enhance the CD3+CD4+/Treg and CD3+CD8+/Treg ratio hence highlighting that this type of therapy alters the immune status toward an anti-tumorigenic environment that curbs regulatory mechanism. This observation was also made with the 1MT treatment despite this type of treatment being rather inefficient in our model. This likely indicates that the decrease in Treg numbers is not sufficient by itself and that other anti-tumorigenic effects play a more relevant role. Such anti-tumorigenic effects may include the significant increase in NK cell numbers that was seen in the group receiving the triple immunotherapy (38).

In conclusion, data presented in this study show that triple immunotherapy, consisting of anti-CTLA-4, MPLA, and 1-MT, is advantageous over other combinations examined. Future studies will include investigations of the mechanisms underlying the survival and tumor progression outcomes described herein. This would encompass examining the phenotypes of the different tumor-infiltrating immune cells in addition to their localization in the tumor. The efficacy of this triple combination in the treatment of other types of tumors will also be tested to examine its applicability to a wider range of cancer types.

## Data Availability Statement

The raw data supporting the conclusions of this article will be made available by the authors, without undue reservation, to any qualified researcher.

## Ethics Statement

The animal study was reviewed and approved by Institutional Animal Care and Use Committee at the American University of Beirut.

## Author Contributions

Conceptualization of the study was by AA. Funding acquisition, methodology, validation, supervision, and manuscript review and editing were done by ER and AA. Data curation, formal analysis, and original draft writing were done by M-AJ. Investigation was carried out by M-AJ, ER, AJ, and AA. All authors read and approved the submitted version of the manuscript.

## Conflict of Interest

The authors declare that the research was conducted in the absence of any commercial or financial relationships that could be construed as a potential conflict of interest.
